# Do Payment Methods Incentivize Screening for Unhealthy Alcohol Use in Ambulatory Care Settings? Evidence from the US National Ambulatory Medical Care Survey

**DOI:** 10.1007/s11414-025-09965-z

**Published:** 2025-09-28

**Authors:** Aryn Z. Phillips, SunJung Yoon

**Affiliations:** 1 Department of Health Policy and Management, School of Public Health, University of Maryland, 4200 Valley Drive, College Park, MD 20742 USA; 2 Department of Health Policy and Administration, School of Public Health, University of Illinois Chicago, 1603 W Taylor Street, Chicago, IL 60612 USA

## Abstract

Despite recommendations, screening for unhealthy alcohol use occurs infrequently in US ambulatory care. One barrier to screening often cited by physicians is misalignment of incentives, but it is unclear which financial arrangements contribute to or alleviate this misalignment. This analysis investigates how determinants of income—both patient care revenue and physician remuneration—encourage or discourage guideline-concordant alcohol screening. Cross-sectional data from the US National Ambulatory Medical Care Survey 2015, 2016, 2018, and 2019 are pooled. Covariate-adjusted logistic regression is used to estimate associations between alcohol screening during visits and variables capturing methods of patient care revenue generation and of physician remuneration. Methods of revenue generation include receipt of revenue from capitation and Medicaid. Methods of physician remuneration include payment by share of practice billings, consideration of productivity and patient satisfaction in determining compensation, and practice ownership. Of 10,607 visits under study, fewer than 3% included screening. In the adjusted model, visits to physicians who received > 25% of patient revenue from capitated payments had higher odds of including screening (adjusted odds ratio (aOR) = 5.94; 95% confidence interval (CI) = 2.33, 15.13) compared with visits to physicians who received less from capitation, as did visits to physicians for whom patient satisfaction surveys impacted compensation (aOR = 3.56; 95% CI = 1.51, 8.37). Payment methods that reward value (e.g., capitation) and patient-centered outcomes (e.g., patient satisfaction), rather than productivity, may promote alcohol screening in US ambulatory care. However, the low rates of screening observed suggest transitioning towards such methods will not be sufficient to achieve optimal screening rates.

**Supplementary Information:**

The online version contains supplementary material available at 10.1007/s11414-025-09965-z.

## Introduction

Unhealthy alcohol use is a leading cause of preventable death in the USA.^1^ This term refers to a wide range of drinking behaviors, such as exceeding the guideline-suggested number of daily or weekly drinks, drinking during pregnancy, and drinking in ways that have a negative impact on other health conditions.^2^ Alcohol use disorder (AUD), a diagnosable condition characterized by the inability to stop or control alcohol use despite social, occupational, or health consequences,^3^ also falls under the banner of unhealthy alcohol use. It is estimated that, in 2010, unhealthy alcohol use cost the US $249 billion.^4^ National data suggest that alcohol was involved in nearly 5 million emergency department visits in 2014^5^ and accounted for over 140,000 deaths per year between 2015 and 2019.^1^

In light of this burden and evidence that brief intervention in primary care can lead to reduced drinking among individuals with unhealthy alcohol use,^6,7^ the US Preventive Services Task Force (USPSTF) has since 1996 recommended alcohol screening and intervention in primary care. Specifically, it recommends that all patients aged 18 and older presenting to primary care be screened for unhealthy alcohol use and that those who report such use receive brief behavioral counseling interventions.^2^ The American Medical Association (AMA) similarly suggests that primary care physicians establish routine alcohol screening processes for all patients and learn to provide brief behavioral counseling. The AMA additionally encourages screening in other medical and surgical specialties in which “undetected alcohol use could affect care.”^8^

Despite these recommendations, alcohol screening remains infrequent in US ambulatory care. Estimates of national screening rates vary widely depending on the data sources, but all suggest that universal screening, let alone universal *high-quality* screening, is not being achieved.^9,10^ Recent research using the Behavioral Risk Factor Surveillance Survey suggests that, among individuals who had a check-up within the last 2 years, 81% were asked about their alcohol consumption. However, only 38% were asked about binge drinking,^9^ a required component of most validated screening tools. Research based on the National Ambulatory Medical Care Survey has found as few as 2.6% of ambulatory care visits included alcohol screening. 

Previous work has identified a host of barriers to screening for unhealthy alcohol use in these settings. Along with such impediments as provider discomfort with discussing alcohol use, inadequate training, and limited resources for screening and referral, a frequently cited barrier is misalignment of incentives.^10–12^ The US health care environment, characterized by fee-for-service reimbursements, high demand for services, and time-constrained physicians, provides few incentives for physicians to screen patients for unhealthy alcohol use.^13,14^ Requirements vary across payors but, among other restrictions, there are limits regarding what types of providers can administer screenings and brief interventions and how long the services must last for them to be eligible for reimbursement.^13^ Even when eligible, reimbursement rates are low and may not be sufficient to cover the cost of the services rendered.^13^

As policy discussions around health care delivery and payment models continue to evolve, a better understanding of this incentive misalignment and, particularly, which financial arrangements may encourage or discourage screening is critical. Previous work has evaluated the introduction of discrete pay-for-performance programs, in which providers are rewarded for conducting alcohol screenings.^12,15–18^ However, such programs are often small in scale and time bound, and studies of these programs may have limited generalizability. To the authors’ knowledge, no studies have considered how methods of payment *typical* in US ambulatory care delivery influence the likelihood of alcohol screening. These methods include those that affect the patient care revenue received by practices as well as those that affect individual physician remuneration (e.g., salary). The objective of this study was to assess the association of such methods of payment with the delivery of guideline-concordant alcohol screening in ambulatory care visits.

### Theoretical Background

#### Patient Care Revenue

The likelihood that screening for unhealthy alcohol use is conducted by an ambulatory care practice may be influenced by the methods by which that practice receives patient care revenue. One such method is whether revenue is received via capitation, in which physician practices are paid a fixed amount to provide care for a patient for a set period. By nature, capitation encourages providing care in ways that can prevent future service utilization, as the practice retains any savings from the capitated payment.^19^ Individuals who have AUD are more likely to experience emergency department visits and other types of costly utilization,^20^ which could perhaps be prevented by screening and subsequent receipt of appropriate intervention. Practices and independent physicians that gain revenue via capitation may, thus, find it cost-effective to invest in robust systems for alcohol screening, brief intervention, and referral to treatment.^13^ Receiving a significant proportion of revenue from capitation also encourages team-based care and more substantial roles for allied health professionals,^21,22^ potentially shifting the responsibility of screening from time-constrained physicians to other staff members.

However, it is also possible that receiving revenue via capitation *discourages* alcohol screening. Given the payment dispersed to the practice remains the same regardless of the services provided, payment via capitation may discourage physicians from providing any additional services.^23,24^ Furthermore, capitation typically motivates physicians to take on a higher volume of patients,^19^ which may result in shorter patient visits and fewer opportunities for alcohol screening.

The amount of revenue received from Medicaid may also influence the likelihood of screening occurring. Resource dependence theory suggests that, in uncertain environments, organizations act in ways that help them manage their relationships with other organizations that control their needed resources (e.g., funding, customers), regardless of whether these actions improve efficiency.^25^ The Centers for Medicare and Medicaid Services (CMS) has increasingly been prioritizing the provision of high-quality substance use-related care to Medicaid beneficiaries,^26^ evidenced by many of its programs for care delivery transformation. Such programs include Medicaid Health Homes, which integrate physical and behavioral health care for individuals with chronic conditions, and the Medicaid Innovation Accelerator Program, which provides technical assistance and resources for care delivery redesign to improve detection and treatment of substance use disorders. Physician practices that receive substantial revenue from Medicaid may participate in these programs in efforts to manage their dependence on CMS. As a result, they may be more likely to have systems in place for alcohol screening. Even if not participating, those that rely heavily on Medicaid revenue may invest in such systems in anticipation of future pressures from CMS.

#### Physician Remuneration

Regardless of whether a physician practice invests in a system for alcohol screening, physician remuneration schemes may shape individual physicians’ likelihood of conducting screening in any given encounter. For instance, being compensated based on one’s share of practice billings may disincentivize screening in comparison to compensation via salary, hourly rate, or other fixed payment. If screening is not billable, or billable but not adequately reimbursable, one might expect physicians who are compensated based on share of billings to forgo screening in favor of spending time on more lucrative services or seeing more patients. The same dynamics are likely to impact physicians for whom the amount of compensation (e.g., salary, bonus, pay rate) is influenced by measures of their productivity.

In addition, the consideration of patient satisfaction in determining a physician’s amount of compensation may influence the likelihood of a physician administering screening. Existing literature suggests this influence could be either positive or negative. From one perspective, alcohol use is a sensitive topic, and previous work suggests some physicians are hesitant to screen patients for unhealthy alcohol use for fear of upsetting the patient.^11,27^ Physicians whose compensation depends on patient satisfaction may feel this concern more acutely and be further unlikely to screen. Alternatively, incorporating patient satisfaction into compensation has been associated with lower odds of physicians reporting that they feel income pressures—that they cannot make appropriate clinical decisions for patients without it impacting pay.^28^ If the use of patient satisfaction in determining compensation alleviates some income pressure, it may foster the development of deeper, trusting relationships with patients, which in turn may promote screening for unhealthy alcohol use.

Finally, ownership of a practice may influence incentives to screen for unhealthy alcohol use. Full or part owners of a practice, who are accountable for a practice’s financial viability and survival, may feel more pressure to forgo screening in favor of billable services.^23,29^

## Methods

### Data

This study used data from the National Ambulatory Medical Care Survey (NAMCS), a nationally representative cross-sectional sample of ambulatory care visits. The survey, administered and made publicly accessible by the National Center for Health Statistics (NCHS), is conducted annually: 1973–1983, 1985, and 1989–present. NAMCS uses a stratified two-stage sampling design. In the first stage, non-federally employed physicians who work in offices (including hospital-owned offices) and are “principally engaged in patient care” are identified from the American Medical Association (AMA) and American Osteopathic Association (AOA) master files, stratified according to region and specialty, and randomly sampled from these strata. The sampling frame excludes physicians who specialize in anesthesiology, pathology, or radiology and physicians who are 85 years and older. In the second stage, the sampled physicians are randomly assigned to a week in the year, and patient visits are randomly sampled from within this week. Physicians complete an induction survey about themselves and their practices. Data collection agents complete a patient record form for each of the visits sampled using information from medical records.^30,31^

This analysis used data from the 2015, 2016, 2018, and 2019 surveys; 2017 survey data were not available. These four years of data contained information on 59,700 patient visits. Following suggestions and protocols that patients be screened once per year,^32–34^ the analysis was restricted to visits by patients who were new to physician practices (negative response to the question “has the patient been seen in your practice before?” *n* = 11,619) and established patients who had not been seen in the past year (response of zero to the question “How many past visits in the last 12 months?” *n* = 4706). These variables were imputed by NCHS if missing. After excluding observations with missing data on variables described below (including answers of “don’t know” or refusal to answer, *n* = 5718; see Supplementary Table 1 for additional detail), the sample included data on 10,607 visits to 1,618 unique physicians. While the research question relates to physician incentives, the analysis was conducted at the visit level, as patient characteristics such as sex, race, ethnicity, age, and presence of other conditions can also impact the likelihood of receiving alcohol screening.^9,10,35^

### Variables

#### Outcome

The outcome of interest was whether the visit included alcohol screening. This information came from patient record forms, in which data collection agents checked boxes denoting the examinations and screenings that occurred during each visit. One such box was for “alcohol misuse screening (includes AUDIT, MAST, CAGE, and T-ACE).”^30^

#### Methods of Patient Care Revenue Generation

Information on patient care revenue came from the physician induction survey. Receipt of revenue from capitation was operationalized using a dichotomous variable, for which a value of one denoted that the physician reported that more than 25% of patient care revenue came from capitation, and a value of zero denoted they reported 25% or less came from capitation. The use of a 25% threshold follows previous literature.^23^ Receiving revenue from Medicaid was operationalized similarly, with a dichotomous variable denoting the physician reported more than 25% of revenue versus 25% or less came from Medicaid.

#### Methods of Physician Remuneration

Information on physician remuneration schemes was also observed in the induction survey. Income being comprised of one’s share of practice billings was measured using a dichotomous variable. When asked which method best describes their basic compensation, physicians who reported they were compensated based on “share of practice billings or workload” or “mix of salary and share of billings or other measures of performance”^30^ received a value of one. Physicians who reported that they were compensated by a fixed salary; shift, hourly, or other time-based payment; or some other method of compensation received a value of zero. The consideration of factors indicative of productivity and of the results of patient satisfaction surveys in compensation were both operationalized using dichotomous variables, with values of one assigned if physicians marked boxes affirming that “factors that reflect [one’s]…own productivity” and the “results of satisfaction surveys from [one’s]…own patients” were “explicitly consider[ed]” in compensation.^30^ They received a value of zero if the box was not marked. Ownership was measured with a binary variable denoting the physician reported being a full or part owner of the practice versus being an employee or contractor.

#### Control Variables

The models included variables for the physician’s specialty, categorized as primary care (e.g., family medicine, internal medicine), surgical care, or other medical care (e.g., dermatology, neurology),^30^ as well as whether the practice was owned by physicians or a physician group; a medical/academic health center, community health center, or other hospital; or an insurance company, health plan, health maintenance organization, or other health corporation. Models also controlled for whether the practice was a solo practice or a partnership of multiple physicians, as evidence suggests providers in multi-physician practices are less likely to order tests than providers in solo practices.^36^ They controlled for a measure of electronic capabilities (i.e., practice had a fully electronic health record versus used all or some paper records), as these capabilities may include decision support or electronic reminders for screening that would increase its likelihood of occurring. They further controlled for the length of the patient visit (15 min or fewer, 16–30 min, or greater than 30 min), given shorter visits are less likely to include additional services. Finally, they controlled for patient sex (male, female), race and ethnicity (mutually exclusive categories of non-Hispanic Black, non-Hispanic White, Hispanic, and non-Hispanic other race), age in years, and number of chronic conditions (zero versus one or more). Length of visit, sex, race, ethnicity, and age were imputed by NCHS if missing.

### Statistical Analyses

First, summary statistics (means and percentages) were estimated for the sample of visits as a whole and by whether alcohol screening was provided, testing for statistically significant differences in means and proportions. Subsequently, a logistic regression model was estimated to test associations between alcohol screening and the variables capturing payment methods, adjusting for the control variables specified. A second model was estimated that included interactions between ownership and receiving revenue from capitation and between ownership and receiving revenue from Medicaid, as ownership may make patient care revenue pressures more salient to a physician.^23^ Both models included year fixed effects. All analyses incorporated the complex survey design and non-response weights using the “svy” command in Stata, which accounts for non-independence of observations within physicians in the variance estimation.^37,38^ Standard errors were estimated using Taylor linearization.

As a sensitivity analysis, the models were re-estimated excluding visits by individuals who were pregnant (*n* = 223) or who had “alcohol misuse, abuse, or dependence” (*n* = 77) at the time of the visit, as documented in the patient record. Screening protocols may vary for these populations, and their inclusion could bias estimates. The models were also re-estimated on a subsample of visits to primary care physicians only (*n* = 2201), given screening may be considered and prioritized differently in primary care compared with other specialties.

This project was deemed not human subjects research by a committee on research ethics and follows the STROBE checklist for cross-sectional studies.

## Results

Table 1 presents descriptive statistics of the sample. Of the 10,607 visits in the sample, over half were incurred by female patients (58.3%, weighted). Over two-thirds of the visits were among non-Hispanic White patients (68.1%, weighted), and 11.0% (weighted) and 13.6% (weighted) were among non-Hispanic Black and Hispanic patients, respectively. The average age was 47 years (standard deviation = 25.5), and over half of patients had one or more chronic conditions (52.0%, weighted).

**Table 1:** Characteristics of ambulatory care visits, *n* = 10,607

	Total		Included alcohol screening			
			No		Yes	
	*N* = 10,607		*N* = 10,465		*N* = 142	
	*N*	%	*N*	%	*N*	%
Patient age (mean ± S.D.)	47.1	± 25.5	46.9	± 25.8	54.4	± 14.0
Patient of female sex	5997	58.3%	5912	58.2%	85	61.4%
Patient race and ethnicity						
Non-Hispanic White	7931	68.1%	7832	68.4%	99	58.6%
Non-Hispanic Black	937	11.0%	925	10.9%	12	15.3%
Hispanic	1209	13.6%	1185	13.3%	24	22.0%
Non-Hispanic other	530	7.3%	523	7.4%	7	4.1%
Patient has 1 + chronic conditions	5558	52.0%	5481	51.5%	77	69.4%
> 25% of patient care revenue from capitation	1078	14.4%	1040	13.4%	38	46.9%*
> 25% of patient care revenue from Medicaid	1652	15.7%	1627	15.6%	25	19.1%
Physician paid with share of practice billings	7080	64.4%	6973	64.4%	107	65.2%
Factors that reflect productivity considered in determining compensation	6615	57.4%	6516	56.8%	99	78.6%*
Patient satisfaction surveys considered in determining compensation	1470	12.9%	1432	12.0%	38	42.3%
Physician is full or part owner in practice	7100	69.6%	7000	69.5%	100	73.4%
Physician specialty						
Primary care	2201	35.6%	2120	34.4%	81	74.2%*
Surgical care	5214	30.9%	5178	31.5%	36	10.1%*
Medical care	3192	33.5%	3167	34.1%	25	15.7%*
Practice owned by						
Physician or physician group	8475	80.5%	8356	80.3%	119	88.4%
Medical/academic health center, community health center, hospital	965	7.5%	943	7.4%	22	11.2%
Insurance company, health plan, HMO, or other health corporation	1167	11.9%	1166	12.3%	1	0.4%*
Solo practice	3677	40.1%	3627	40.5%	50	24.5%
Fully electronic health records	8036	74.1%	7908	73.6%	128	91.5%*
Time spent in visit						
0–15 min	4109	34.0%	4080	34.1%	29	30.4%
16–30 min	4657	46.6%	4570	46.4%	87	53.4%
> 30 min	1841	19.4%	1815	19.5%	26	16.2%

*S.D.*, standard deviation; *HMO*, health maintenance organization; *EHR*, electronic health record. * Denotes statistically significant difference (*p* < 0.05) when compared to no alcohol screening in test of means or proportions. Estimates of means, standard deviations, and percentages, and tests of means and proportions incorporate complex survey design and are weighted for non-response

A minority of visits occurred with physicians who reported that more than 25% of revenue came from capitation and from Medicaid (14.4 and 15.7%, respectively, weighted). Over half of visits were with physicians who were compensated with a share of practice billings versus with salary, etc. (64.4%, weighted) and with physicians who reported that productivity was used to determine their compensation (57.4%, weighted). Only 12.9% (weighted) of visits were with physicians who reported that patient satisfaction surveys were used to determine compensation. Most visits occurred with physicians who were full or part owners of the practice (69.6%, weighted).

Only 142 of the 10,607 visits under study (2.9%, weighted) included alcohol screening. There were significant differences between visits that did and did not include screening (see Table 1). Compared with visits that did not include screening, a significantly higher proportion of visits that did include screening occurred with physicians for whom more revenue came from capitation, with physicians for whom productivity was considered in compensation, with primary care physicians, and with physicians with fully electronic health records. A significantly smaller proportion of visits with screening were with physicians in a practice owned by an insurance company, health plan, health maintenance organization, or other health corporation.

In the covariate-adjusted logistic model, the odds of screening occurring were higher in visits to physicians for whom more than 25% of revenue came from capitation compared to the odds of screening occurring in visits to physicians for whom less revenue came from capitation, adjusting for covariates (adjusted odds ratio (aOR) = 5.94; 95% confidence interval (CI) = 2.33, 15.13). Additionally, the odds of screening occurring in visits to physicians for whom results of patient satisfaction surveys are used to determine compensation were higher than the odds of screening occurring in visits to physicians for whom patient satisfaction is not considered (aOR = 3.56; 95% CI = 1.51, 8.37). There was no evidence suggesting any of the other payment methods were linked with screening, nor was there evidence of significant interactions between ownership and patient care revenue generation methods (Table 2).

**Table 2:** Association of alcohol screening with methods of revenue generation and physician remuneration, *n* = 10,607

	Model 1	Model 2
	Odds ratio	Odds ratio
	*P*-value	*P*-value
	(95% CI)	(95% CI)
> 25% of patient care revenue from capitation	**5.94**	3.18
	** < 0.001**	0.24
	**(2.33–15.13)**	(0.47–21.52)
> 25% of patient care revenue from Medicaid	1.04	1.53
	0.95	0.66
	(0.36–2.97)	(0.23–10.17)
Paid via share of practice billings	1.30	1.41
	0.53	0.36
	(0.57–2.99)	(0.68–2.94)
Factors that reflect productivity considered in compensation	1.23	1.25
	0.72	0.70
	(0.39–3.85)	(0.40–3.91)
Patient satisfaction surveys considered in compensation	**3.56**	**3.65**
	**0.004**	**0.002**
	**(1.51–8.37)**	**(1.59–8.38)**
Physician is full or part owner of practice	1.50	1.19
	0.55	0.77
	(0.40–5.59)	(0.37–3.86)
Physician is full or part owner *x* > 25% of patient care revenue from capitation	-	2.26
	-	0.47
	-	(0.25–20.50)
Physician is full or part owner *x* > 25% of patient care revenue from Medicaid	-	0.57
	-	0.60
	-	(0.07–4.64)

Table presents odds ratios, *p*-values, and 95% confidence intervals (CI) from two logistic regression models. Model 1 is estimated without interactions between physician ownership and revenue generation determinants, and Model 2 is estimated with interactions. Both models additionally control for physician specialty, practice ownership, whether the practice is a solo or multi-physician practice, practice electronic capabilities, length of patient visit, and patient sex, race and ethnicity, age, and chronic condition burden (results not shown for brevity; available from authors upon request). Both models include year fixed effects, incorporate the complex survey design, and are weighted to account for non-response. Boldface indicates statistical significance at the *p* < 0.05 level

Only 3.0% (weighted) of visits include screening when the sample is limited to those by individuals who were not pregnant or who had not been diagnosed with alcohol misuse, abuse, or dependence. The model estimated on this sample similarly suggested higher odds of screening in visits to physicians who received more than 25% of patient revenue from capitation (aOR = 5.94; 95% CI = 2.30, 15.30) and in visits to physicians for whom patient satisfaction was considered in compensation (aOR = 3.73; 95% CI = 1.58, 8.79), adjusting for covariates (see Supplementary Table 2). When incorporated, interactions between physician ownership and methods of patient care revenue generation were not significantly associated with screening (results not shown).

A higher percentage of visits included screening when the sample was limited to visits to primary care physicians (6.1%, weighted). In this subsample, receipt of revenue from capitation was again linked to higher odds of screening occurring (aOR = 9.77; 95% CI = 2.79, 34.25), but the use of patient satisfaction surveys in determining compensation was only significantly associated with odds of screening at the 0.10 level (see Supplementary Table 3). This model additionally suggested that ownership was positively associated with higher odds of screening, although the confidence interval was wide (aOR = 7.27; 95% CI = 1.35, 39.06). Again, there was no evidence that ownership significantly interacted with revenue generation methods (results not shown).

## Discussion

This analysis finds that, in a national sample of ambulatory care visits, the provision of screening for unhealthy alcohol use was associated with ways in which both patient care revenue is generated and ways in which individual physicians are paid. In terms of revenue generation, the evidence suggests that visits with physicians who received more than 25% of patient revenue via capitation had higher odds of including screening compared to visits with physicians who did not receive as much revenue in this manner. While it remains that the relationship may be endogenous, receipt of revenue via capitation may promote screening, perhaps by encouraging the treatment of unhealthy alcohol use to avert subsequent high-cost utilization. It also may encourage team-based care in ways that relieve time-constrained physicians of screening.

Regarding physician remuneration, this study finds that visits with physicians for whom patient satisfaction surveys were considered in compensation had higher odds of including alcohol screening. Previous research suggests the use of patient satisfaction in compensation may encourage alcohol screening by relieving some income pressures that may hinder screening. However, there was no evidence of a relationship between productivity-based compensation and odds of screening in this analysis, suggesting such income pressures may not influence screening administration. Additional research will be necessary to understand the mechanisms underlying this result. Some of the associations observed may be due to physician selection. Physicians select into remuneration schemes,^39,40^ and physicians who opt into positions in which their compensation is tied to patient satisfaction may differ from those who do not in how they provide patient care broadly and alcohol-related services specifically. Previous work, however, has found that remuneration schemes have incentive effects even when accounting for physician selection.^41^ It is worth noting that this association was only significant at the 0.10 level in the sample limited to primary care visits, possibly due to the smaller size of this sample or to differences in the training, workflows, priorities, etc., of primary care physicians compared with other physicians.

A major strength of this study is the use of a nationally representative sample of patient visits, which provides some generalizability to US ambulatory care visits by new patients or established patients without a visit in the prior year during the years under observation. The use of multiple years of data also provides a large enough sample size to observe and make inferences about what is observed to be a rare outcome. Nonetheless, the study is subject to several limitations. First, the conclusions rely on physicians’ reports about their practices, in which there may be inaccuracies; some physicians, for instance, may not know precisely how much patient care revenue comes from various sources. Overestimates or underestimates could bias the results in either direction. Relatedly, there is considerable missingness in the data, the majority of which is in the variables measuring receipt of revenue from capitation and Medicaid. The authors opted for casewise deletion rather than imputation to avoid potential bias,^42^ but it remains that the estimates and their variability may be impacted by this analytic choice. This pattern in the missingness suggests that visits to physicians with more thorough knowledge of their practices’ financial arrangements (perhaps in leadership positions) may be disproportionately represented in this sample. In addition, information on alcohol screening is sourced from patient medical records, and screening, particularly brief screening, may not be documented in these records or may be documented inconsistently. Either scenario could introduce bias into the estimates. Further, as noted, the data do not allow for observation of the mechanisms underlying the associations identified. Understanding these mechanisms is critical for developing policies that will improve screening rates, but this work contributes important preliminary findings that can inform future investigations to uncover these mechanisms. Finally, the rarity of screening, in combination with the chosen predictors, results in wide confidence intervals for many estimates, particularly in the sub-analyses limited to visits to primary care physicians. As such, the authors give less credence to the magnitude of these estimates than they do their direction as they interpret the results. The results should be corroborated with future studies.

## Implications for Behavioral Health

The findings regarding capitation and patient satisfaction, taken together and with the lack of evidence for other associations, indicate that methods of payment that reward value and patient-centered outcomes, rather than productivity, may help incentivize alcohol screening. Policies that encourage participation in payment and delivery models that prioritize such outcomes may result in improvements in the provision of alcohol-related care. However, it is important to emphasize that less than 3% of the visits in this sample included alcohol screening. This estimate is a dramatic departure from national estimates of screening from other sources, particularly those that rely on survey responses from the patient perspective, which suggest 70–83% of individuals receive some alcohol assessment.^9,35^ These estimates may be different due to inaccurate patient recall or because screenings, as noted, may not be recorded in medical records. Even if a substantial underestimate, this estimate, which aligns with those of others quantifying alcohol screening in NAMCS data,^10^ speaks to the volume of work that remains to achieve universal high-quality screening. Transitioning towards payment models that reward value and patient satisfaction may be helpful in this effort but, in all probability, will be insufficient to achieve such screening in ambulatory care settings. New payment models will likely need to be accompanied by training, technical support, funding, and other resources that will facilitate the adoption and thorough implementation of screening processes. Future research should investigate how the provision of these resources, in combination with payment models that prioritize value and satisfaction, may improve screening rates. If effective, funding agencies that support screening implementation and clinical practices seeking to improve screening rates may want to consider both in tandem.

## Conclusion

This study investigates how methods of payment, both in terms of how patient care revenue is generated and in terms of physician remuneration, are linked to screening for unhealthy alcohol use during ambulatory care visits. It finds that, in a national sample of visits, the odds of screening occurring were higher among visits to physicians who received more revenue via capitation and among visits to those for whom compensation was at least partially dependent on patient satisfaction. These findings suggest that payment and delivery system innovations that reward value and patient-centered outcomes, rather than quantity and productivity, may help improve the rates of alcohol screening and the provision of alcohol-related care in general.

## Supplementary Information

Below is the link to the electronic supplementary material.

Supplementary file1 (DOCX 30 KB)

## Data Availability

Data are from the US Centers for Disease Control and Prevention National Center for Health Statistics and are publicly available at the following website: https://www.cdc.gov/nchs/namcs/documentation/index.html.
