# Supplementary material for: Do Payment Methods Incentivize Screening for Unhealthy Alcohol Use in Ambulatory Care Settings? Evidence from the US National Ambulatory Medical Care Survey
Source: J Behav Health Serv Res. 2025 Sep 28;53(1):3–18. doi: 10.1007/s11414-025-09965-z (PMC12876462; doi:10.1007/s11414-025-09965-z)
Supplement: Supplementary file 1 — Supplementary file1 (DOCX 30 KB) [file 11414_2025_9965_MOESM1_ESM.docx]

Supplementary Material

| Supplementary Table 1. Missing data by variable. | | | | |
| --- | --- | --- | --- | --- |
| Variable | Number of observations missing data | | | |
|  | Total missing | Blank | Don’t know/ unknown | Refused to answer |
| Revenue received from capitation | 4,281 | 4,281 | 0 | 0 |
| Revenue received from Medicaid | 3,128 | 3,111 | 17 | 0 |
| Paid with share of practice billings (vs. salary, etc.) | 1,040 | 0 | 871 | 169 |
| Ownership (full or part owner vs. employee or contractor) | 92 | 0 | 92 | 0 |
| Who owns the practice (physician/physician group, medical/academic health center, etc.) | 600 | 530 | 70 | 0 |
| Solo vs. non-solo practice | 15 | 0 | 15 | 0 |
| Electronic health record capabilities | 55 | 0 | 52 | 3 |
| Patient's number of chronic conditions | 318 | 318 | 0 | 0 |
| Total observations missing some data | 5,718 |  | | |
| Total observations with complete data | 10,607 |  | | |
| Note. “Total missing” is sum of “Blank,” “Don’t know/unknown,” and “Refused to answer” for each variable. Some observations are missing data on multiple variables; as a result, the number of observations missing for each variable do not sum to total observations with missing data. | | | | |

| Supplementary Table 2. Association of alcohol screening with methods of revenue generation and physician remuneration, excluding patients who are pregnant or who were previously diagnosed with alcohol use disorder, n=10,307. | |
| --- | --- |
|  | Odds Ratio |
|  | P-value |
|  | (95% CI) |
| Percent of patient care revenue from capitation |  |
| 0-25% | Ref. |
| >25% | **5.936** |
|  | **<0.001** |
|  | **(2.303 - 15.299)** |
| Percent of patient care revenue from Medicaid |  |
| 0-25% | Ref. |
| >25% | 1.129 |
|  | 0.821 |
|  | (0.394 - 3.235) |
| Paid via share of practice billings |  |
| No | Ref. |
| Yes | 1.354 |
|  | 0.482 |
|  | (0.581 - 3.155) |
| Factors that reflect productivity considered in determining compensation |  |
| No | Ref. |
| Yes | 1.139 |
|  | 0.821 |
|  | (0.370 - 3.505) |
| Patient satisfaction surveys considered in determining compensation |  |
| No | Ref. |
| Yes | **3.732** |
|  | **0.003** |
|  | **(1.584 - 8.793)** |
| Physician ownership in practice |  |
| Employee or contractor | Ref. |
| Full or part owner of practice | 1.510 |
|  | 0.550 |
|  | (0.391 - 5.827) |
| Physician specialty |  |
| Primary care | Ref. |
| Surgical care | **0.121** |
|  | **<0.001** |
|  | **(0.042 - 0.351)** |
| Medical care | **0.254** |
|  | **0.011** |
|  | **(0.088 - 0.733)** |
| Practice owned by |  |
| Physician or physician group | Ref. |
| Medical/academic health center, community health center, or hospital | 1.652 |
|  | 0.514 |
|  | (0.366 - 7.466) |
| Insurance company, health plan, HMO, or other health corporation. | **0.007** |
|  | **<0.001** |
|  | **(0.001 - 0.091)** |
| Type of practice |  |
| Solo | Ref. |
| Multi-physician | 1.253 |
|  | 0.689 |
|  | (0.415 - 3.783) |
| EHR capabilities |  |
| All or partially paper | Ref. |
| Fully electronic | 3.338 |
|  | 0.072 |
|  | (0.899 - 12.396) |
| Time spent in visit |  |
| 0-15 minutes | Ref. |
| 16-30 minutes | 1.840 |
|  | 0.097 |
|  | (0.895 - 3.782) |
| >30 minutes | 1.561 |
|  | 0.323 |
|  | (0.646 - 3.773) |
| Patient sex |  |
| Female | Ref. |
| Male | 0.948 |
|  | 0.860 |
|  | (0.523 - 1.719) |
| Patient race and ethnicity |  |
| Non-Hispanic White | Ref. |
| Non-Hispanic Black | 1.003 |
|  | 0.992 |
|  | (0.517 - 1.947) |
| Hispanic | **2.497** |
|  | **0.013** |
|  | **(1.213 - 5.140)** |
| Non-Hispanic Other | 0.579 |
|  | 0.506 |
|  | (0.115 - 2.905) |
| Patient age | **1.022** |
|  | **0.002** |
|  | **(1.008 - 1.035)** |
| Patient has 1+ chronic condition |  |
| No |  |
| Yes | 1.401 |
|  | 0.248 |
|  | (0.791 - 2.481) |
| Note. Table presents odds ratios, p-values, and 95% confidence intervals (CI) from logistic regression. The model additionally includes year fixed effects, incorporates the complex survey design, and is weighted to account for non-response.  Ref.=Reference category. HMO=health maintenance organization. EHR=electronic health record. Boldface indicates statistical significance at p<0.05 level. | |

| Supplementary Table 3. Association of alcohol screening with methods of revenue generation and physician remuneration, in primary care visits only, n=2,201. | |
| --- | --- |
|  | Odds Ratio |
|  | P-value |
|  | (95% CI) |
| Percent of patient care revenue from capitation |  |
| 0-25% | Ref. |
| >25% | **9.771** |
|  | **<0.001** |
|  | **(2.787 - 34.249)** |
| Percent of patient care revenue from Medicaid |  |
| 0-25% | Ref. |
| >25% | 0.630 |
|  | 0.397 |
|  | (0.215 - 1.841) |
| Paid via share of practice billings |  |
| No | Ref. |
| Yes | 1.015 |
|  | 0.967 |
|  | (0.494 - 2.087) |
| Factors that reflect productivity considered in determining compensation |  |
| No | Ref. |
| Yes | 1.164 |
|  | 0.861 |
|  | (0.214 - 6.317) |
| Patient satisfaction surveys considered in determining compensation |  |
| No | Ref. |
| Yes | 2.735 |
|  | 0.071 |
|  | (0.917 - 8.153) |
| Physician ownership in practice |  |
| Employee or contractor | Ref. |
| Full or part owner of practice | **7.265** |
|  | **0.021** |
|  | **(1.351 - 39.055)** |
| Practice owned by |  |
| Physician or physician group | Ref. |
| Medical/academic health center, community health center, or hospital | **7.637** |
|  | **0.029** |
|  | **(1.235 - 47.232)** |
| Insurance company, health plan, HMO, or other health corporation. | **0.024** |
|  | **0.009** |
|  | **(0.001 - 0.388)** |
| Type of practice |  |
| Solo | Ref. |
| Multi-physician | 2.012 |
|  | 0.447 |
|  | (0.332 - 12.201) |
| EHR capabilities |  |
| All or partially paper | Ref. |
| Fully electronic | **9.579** |
|  | **0.007** |
|  | **(1.835 - 50.012)** |
| Time spent in visit |  |
| 0-15 minutes | Ref. |
| 16-30 minutes | 1.394 |
|  | 0.430 |
|  | (0.611 - 3.181) |
| >30 minutes | 1.651 |
|  | 0.304 |
|  | (0.634 - 4.300) |
| Patient sex |  |
| Female | Ref. |
| Male | 0.915 |
|  | 0.820 |
|  | (0.426 - 1.965) |
| Patient race and ethnicity |  |
| Non-Hispanic White | Ref. |
| Non-Hispanic Black | 0.782 |
|  | 0.493 |
|  | (0.387 - 1.581) |
| Hispanic | 2.254 |
|  | 0.084 |
|  | (0.896 - 5.669) |
| Non-Hispanic Other | 1.197 |
|  | 0.841 |
|  | (0.207 - 6.928) |
| Patient age | **1.023** |
|  | **<0.001** |
|  | **(1.011 - 1.035)** |
| Patient has 1+ chronic conditions |  |
| No | Ref. |
| Yes | 1.289 |
|  | 0.490 |
|  | (0.627 - 2.648) |
| Note. Table presents odds ratios, p-values, and 95% confidence intervals (CI) from logistic regression. The model additionally includes year fixed effects, incorporates the complex survey design, and is weighted to account for non-response.  Ref.=Reference category. HMO=health maintenance organization. EHR=electronic health record. Boldface indicates statistical significance at p<0.05 level. | |
